# *In Silico* Validation of OncoOrigin: An Integrative AI Tool for Primary Cancer Site Prediction with Graphical User Interface to Facilitate Clinical Application

**DOI:** 10.3390/ijms26062568

**Published:** 2025-03-13

**Authors:** Petar Brlek, Luka Bulić, Nidhi Shah, Parth Shah, Dragan Primorac

**Affiliations:** 1 St. Catherine Specialty Hospital, 10000 Zagreb, Croatia; luka.bulic0302@gmail.com (L.B.);; 2 School of Medicine, Josip Juraj Strossmayer University of Osijek, 31000 Osijek, Croatia; 3 Dartmouth Health, Lebanon, NH 03766, USA; 4 Eberly College of Science, The Pennsylvania State University, State College, PA 16802, USA; 5 School of Medicine, University of Split, 21000 Split, Croatia; 6 The Henry C. Lee College of Criminal Justice and Forensic Sciences, University of New Haven, New Haven, CT 06516, USA; 7 Regiomed Kliniken, 96450 Coburg, Germany; 8 School of Medicine, University of Rijeka, 51000 Rijeka, Croatia; 9 Faculty of Dental Medicine and Health, Josip Juraj Strossmayer University of Osijek, 31000 Osijek, Croatia; 10 School of Medicine, University of Mostar, 88000 Mostar, Bosnia and Herzegovina; 11 National Forensic Sciences University, Gandhinagar 382007, India

**Keywords:** cancer genomics, cancers of unknown primary origin, machine learning, artificial intelligence, tumor genetic profiling

## Abstract

Cancers of unknown primary (CUPs) represent a significant diagnostic and therapeutic challenge in the field of oncology. Due to the limitations of current diagnostic tools in these cases, novel approaches must be brought forward to improve treatment outcomes for these patients. The objective of this study was to develop a machine-learning-based software for primary cancer site prediction (OncoOrigin), based on genetic data acquired from tumor DNA sequencing. By design, this was an *in silico* diagnostic study, conducted using data from the cBioPortal database (accessed on 21 September 2024) and several data processing and machine learning Python libraries. The study involved over 20,000 tumor samples with information on patient age, sex, and the presence of genetic variants in over 600 genes. The main outcome of interest was machine-learning-based discrimination between cancer site classes. Model quality was assessed by training set cross-validation and evaluation on a segregated test set. Finally, the optimal model was incorporated with a graphical user interface into the OncoOrigin software. Feature importance for class discrimination was also determined on the optimal model. Out of the four tested machine learning estimators, the XGBoostClassifier-based model proved superior in test set evaluation, with a top-2 accuracy of 0.91 and ROC-AUC of 0.97. Unlike other machine learning models published in the literature, OncoOrigin stands out as the only one integrated with a graphical user interface, which is crucial for facilitating its use by oncology specialists in everyday clinical practice, where its application and implementation will have the greatest value in the future.

## 1. Introduction

Precision medicine has revolutionized therapy protocols in oncology, enabling the implementation of targeted treatments based on specific molecular biomarkers of tumors [1]. Advanced molecular biology methods, such as whole genome sequencing (WGS) and whole exome sequencing (WES) of tumor tissue, allow for the comprehensive molecular analysis of tumors and the application of therapies that act on specific molecular targets within tumor cells [2]. A cornerstone of this treatment approach is molecular tumor profiling, which involves genetic analysis of formalin-fixed, paraffin-embedded tumor tissue and/or circulating tumor DNA (ctDNA) derived from liquid biopsy samples [3]. The analysis of ctDNA from a patient’s blood represents a significant advancement in oncology, particularly in cases where traditional tissue biopsy is not feasible. Liquid biopsy is a non-invasive method that enables real-time monitoring of tumor evolution and therapeutic response, further enhancing diagnostic accuracy and treatment personalization. These advancements in oncology diagnostics lay the foundation for the introduction of molecular classification of malignancies to guide targeted therapy, particularly in cases of cancer of unknown primary origin.

Cancers of unknown primary (CUPs) make up 5% of all malignant conditions worldwide. They are characterized by their aggressiveness, fast progression, and poor prognosis with alarmingly high mortality rates. Up to 84% of patients have fatal outcomes within the first year after diagnosis and represent some of the most diagnostically and therapeutically challenging cases in the field of oncology [4,5]. Additionally, unknown primary malignancies significantly limit the options for surgical treatment and radiotherapy, while protocols for chemotherapy, immunotherapy, and other targeted therapies cannot be determined without identifying the primary tumor site. In such cases, patients are most often treated with non-specific therapies that are not tailored to a particular tumor type, highlighting the critical role of molecular profiling in guiding personalized therapy protocols in oncology [6]. Traditional diagnostic methods, such as immunohistochemistry using specific markers, have limited diagnostic power, especially in poorly differentiated tumors, and are often not sufficient for primary site verification [4,5].

Tumor molecular profiling with novel next-generation sequencing (NGS) methods, such as WGS, has opened up the possibility of detecting all potential germline and somatic genetic alterations in tumor tissue. This enables more precise molecular classification of tumors and their personalized treatment [7]. Scientific efforts have been made to use genetic profiling for precise molecular classification of tumors. One such example is using single-cell transcriptomics and spatial proteomics for the purpose of improving treatment outcomes [8]. Additionally, WES or WGS of paired tumor and normal tissue enable the detection of tumor-specific neoantigens, opening new possibilities for the development of personalized cancer vaccines [9].

In recent years, machine learning has become a robust tool in biomedical research due to its ability to efficiently process large quantities of data [10]. Certain studies have endeavored to combine genetic testing and machine learning with the purpose of primary site identification [11]. Additionally, machine learning algorithms have shown great potential in the identification of tumor-specific neoantigens based on genomic and transcriptomic data, contributing to the development of personalized cancer vaccines [12]. These vaccines have demonstrated encouraging results, particularly in tumors with poor prognoses, such as glioblastoma [13]. Although these approaches have shown promising results, further research and validation are necessary before their widespread adoption in clinical practice.

The aim of our study was to train a primary cancer site predictor by combining genetic data from over 20,000 tumor samples and different machine learning algorithms. Following evaluation, the optimal model could be fitted into a simple software application that would allow for easy practical implementation. Such a tool could offer a new and robust diagnostic option for CUP cases and potentially significantly improve treatment outcomes for these patients, and in the future, enable the detection of the primary tumor site based on the analysis of liquid biopsies without the need for invasive and harmful medical procedures.

## 2. Results

### 2.1. Data Preprocessing

After data extraction from the cBioPortal database had been completed, data on sex, age at time of sequencing, and genetic variant presence for 665 genes across 21,016 patient tumor samples were selected (Supplemental Table S1). Elimination of uniformly distributed genes produced the database for the modeling pipeline, containing the features of sex, age, and genetic variant presence for 566 genes and 20,710 samples. After feature selection, 345 genetic features remained in the database, along with the sex and age features (Supplemental Table S2). Class frequencies in the output vector were 0.14, 0.09, 0.08, 0.22, 0.22, 0.02, 0.07, 0.11, 0.02, and 0.02 for colorectal cancer, ovarian cancer, melanoma, breast cancer, lung cancer, thyroid cancer, prostate cancer, hepatobiliary/pancreatic cancer, bladder cancer, and endometrial cancer respectively.

### 2.2. Model Evaluation

After feature selection, hyperparameter optimization, and model fitting were complete, evaluation metrics were determined for each of the four estimators. This included metrics received from training set cross-validation and evaluation based on the test set (Table 1).

The results demonstrated the XGBC model to be the superior model in both training set cross-validation and test set evaluation. The CBC model showed only slightly poorer performance metrics, while the ETC and RFC models showed significantly poorer performance metrics. Interestingly, the ETC model, while poorer in overall quality, showed the highest sensitivity values for ovarian cancer, thyroid cancer, bladder cancer, and endometrial cancer.

### 2.3. Class-Specific Evaluation and Feature Analysis

**Figure 1:**
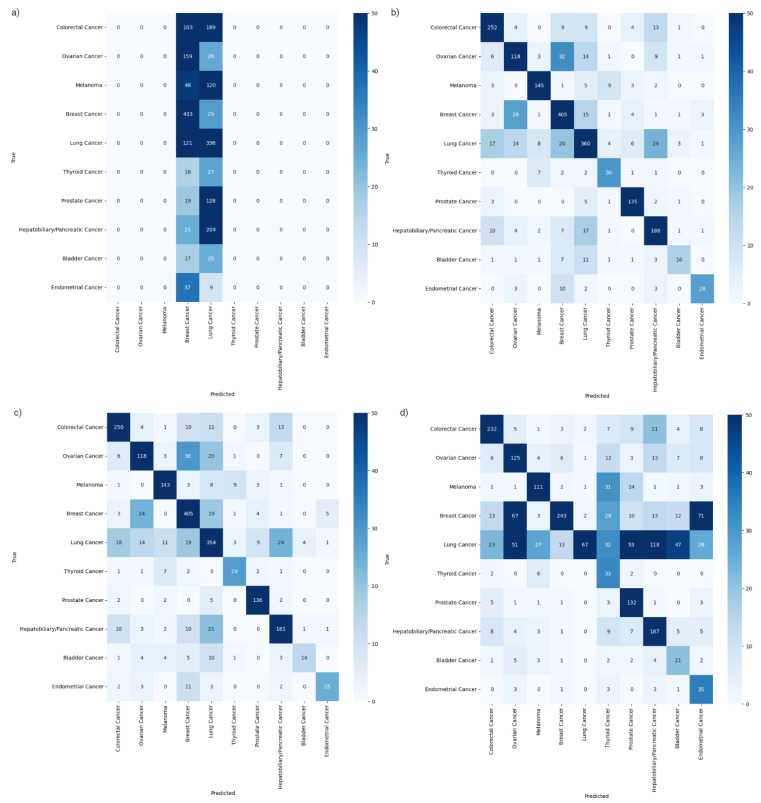
Confusion matrices of the RFC model (**a**), XGBC model (**b**), CBC model (**c**), and ETC model (**d**). These matrices demonstrate results of model performance on the segregated test set.

Confusion matrices were determined for each of the models to better evaluate how each class impacted the overall predictive power (Figure 1).

**Figure 2:**
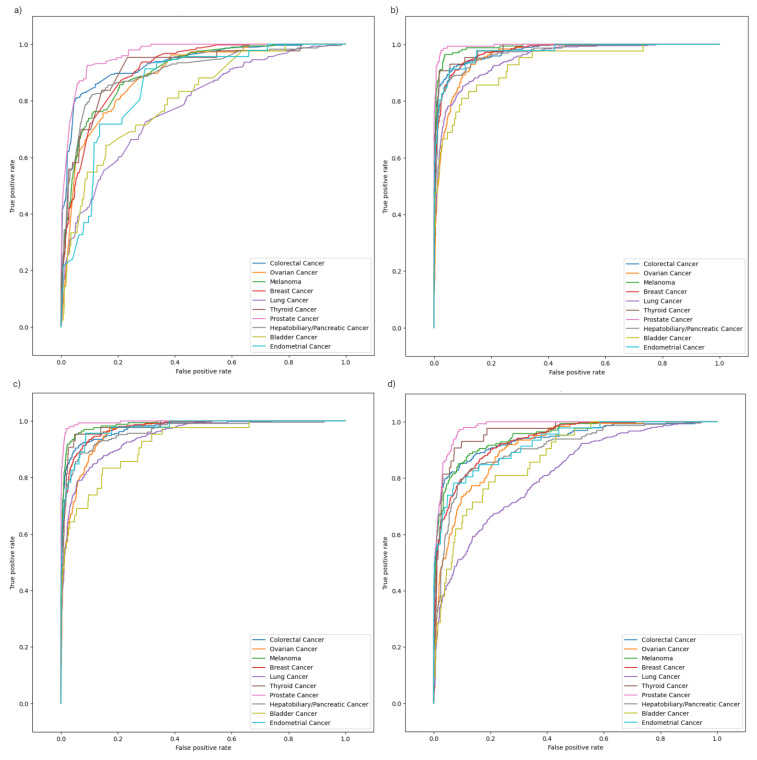
Class-specific ROC analysis curves for the RFC model (**a**), XGBC model (**b**), CBC model (**c**), and ETC model (**d**). These plots demonstrate the results of model performance on the segregated test set.

The RFC confusion matrix showed that the model predicted almost all samples as either breast cancer or lung cancer. The ETC confusion matrix showed a somewhat better result, with a higher ability to detect less represented classes, but a poor ability to detect the lung cancer class. Finally, the XGBC and CBC confusion matrices had a very similar layout. Hotspots were noticed in both fields pertaining to breast cancer and ovarian cancer, indicating a difficulty in differentiating between the two. ROC curves were in line with the model metrics (Figure 2).

**Figure 3:**
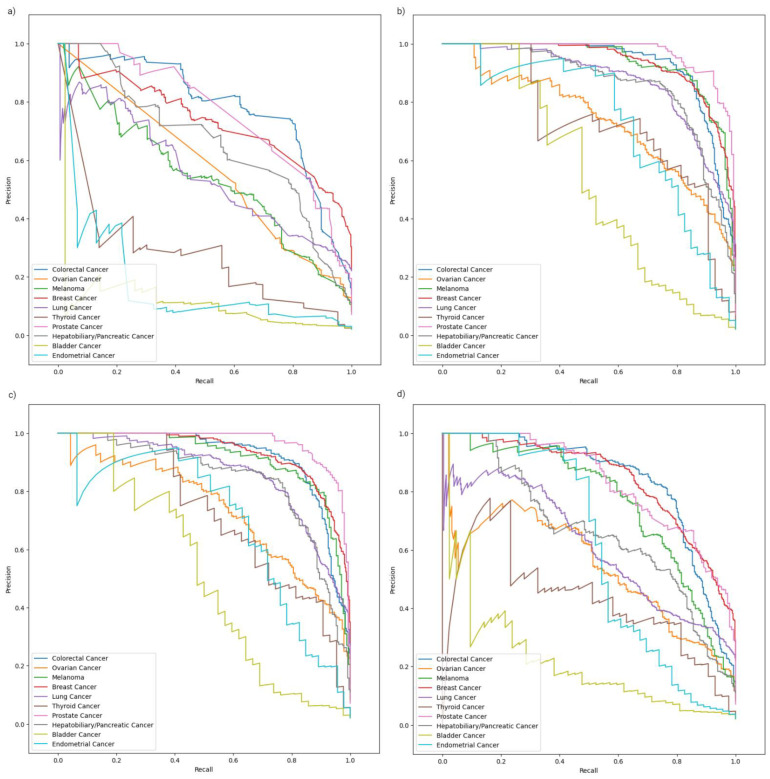
Class-specific precision–recall analysis curves for the RFC model (**a**), XGBC model (**b**), CBC model (**c**), and ETC model (**d**). These plots demonstrate results of model performance on the segregated test set.

In terms of class, the bladder cancer and lung cancer ROC curves visibly stood out with lower AUCs in all models, while the prostate cancer ROC curves were consistently superior across all models. A similar trend was noted in the precision–recall curves (Figure 3).

**Figure 4:**
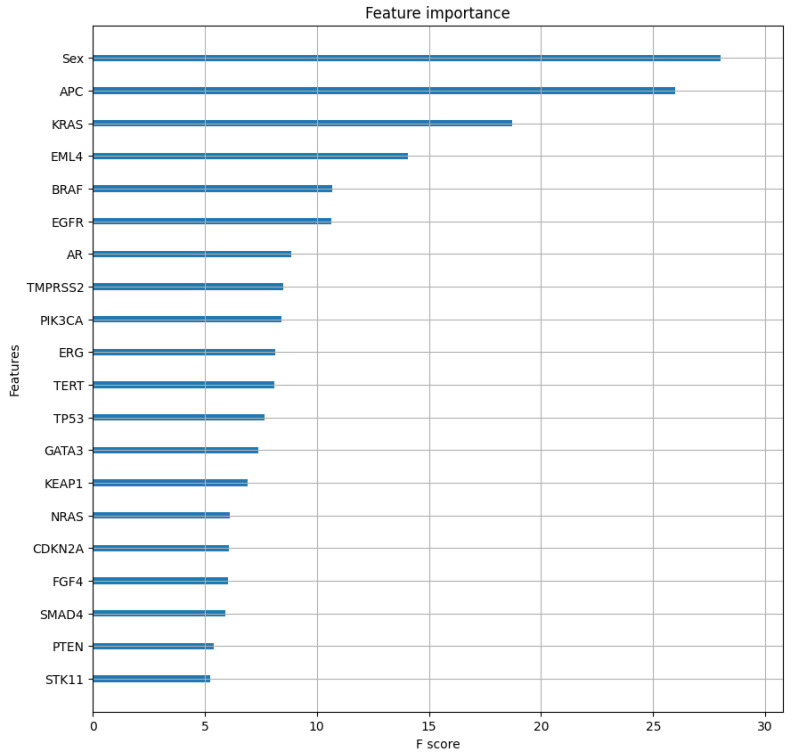
Feature importance analysis of the top 20 features based on the optimal XGBC model.

The feature importance analysis revealed the top 20 features for primary site prediction (Figure 4).

The highest importance score was calculated for the patient sex feature, followed by gene alterations in *APC* and *KRAS*.

## 3. Discussion

### 3.1. Software Quality Evaluation and Comparison

After evaluation and interpretation of the produced results, the XGBoostClassifier was identified as the optimal estimator for OncoOrigin, with an accuracy of 0.81, top-2 accuracy of 0.91, F1-score of 0.81, PR-AUC score of 0.88, and ROC-AUC score of 0.97. As the software provides the user with not only a prediction but also insight into the likelihood of the other classes, clinicians will always be informed whether a second class is highly likely. As this can be clinically actionable information, we deemed the top-2 accuracy metric relevant for our model. When observing specific classes, sensitivity values ≥0.79 were found for breast cancer, lung cancer, colorectal cancer, prostate cancer, melanoma, and hepatobiliary/pancreatic cancer, while the other classes had lower sensitivity values. This trend generally corresponds to the quantitative representation of each class in the total population sample, determined by frequency analysis. Where class specificity values are concerned, all values were ≥0.95.

The performance metrics of our model can be compared to other relevant literature for this context. In the paper published by Moon I et al., the authors also proposed a machine learning model for primary cancer site detection [11]. While the source of data, number of samples used for training and testing, and feature definition and representation were different, the authors reported a weighted F1-score of 0.784 for classification based on 22 cancer types and an F1-score of 0.806 for classification based on 13 cancer types. Our OncoOrigin model measures up to these metrics with a weighted F1-score of 0.81 for classification based on 10 cancer sites. Furthermore, while the authors used different feature selection and hyperparameter optimization methods, they too used the XGBoostClassifier algorithm as the classification estimator. In an article by Bicakci N, the effectiveness of the F-18 FDG PET-CT scan in CUP cases was evaluated [14]. The primary tumor was correctly determined in 41% of patients. The rest of the patients either did not have matching PET-CT or histopathology findings, or failed to have their primary tumor differentiated by both methods. Another study, published by Lu MY et al., constructed a deep learning model to identify primary cancer sites based on histopathological image processing [15]. In their article, the authors reported their model achieved an accuracy of 0.834 when evaluated on the segregated test set. Once again, this is comparable with the 0.81 accuracy that OncoOrigin achieved on our segregated test set.

Unlike other machine learning models published in the literature, OncoOrigin stands out as the only one integrated with a graphical user interface. Normally, interactions with developed machine learning models require a background in AI programming and data science. However, for clinicians and other medical experts, this might be an impractical skill set to acquire and maintain [16]. For this reason, a simple user interface that communicates data between the model and the user greatly facilitates its potential for implementation in clinical practice.

### 3.2. Software Limitations

While quality results were achieved in the evaluation of OncoOrigin, the model does have several limitations. Firstly, the model has a total of 347 input features, the values of which should be known upon using the software to receive a prediction with an accuracy level corresponding to the one presented in this article. This entails the sequencing of 345 genes, which may be challenging, depending on the available technology for any given user. Secondly, due to the format of the extracted data, the model was trained with age values ranging from 18 to 89. For this reason, the age input in the software was limited to this range. Thirdly, while the model did use data combined from multiple studies, these data were still extracted from a single source, which leaves potential for a certain level of data bias and risk of overfitting. Another limitation regarding the data is underrepresentation of certain cancer sites, which likely negatively impacted the ability of the model to identify them. This was not corrected through reweighting or resampling, as the more prevalent classes are significant for CUP identification. Finally, the model is limited to predicting primary sites among the ten cancer site classes included in the study. Where lung cancer is concerned, the small cell and non-small cell cancer subtypes were combined into one class. This was done to improve overall identification of lung cancer sites. However, it does present a drawback as small cell and non-small cell lung cancers are quite different.

### 3.3. Feature Importance Analysis Implications

Aside from clinical utility in terms of primary cancer site prediction, the OncoOrigin feature analysis might also offer interesting insights. The feature of greatest significance was sex, which is to be expected, as it allows for easy elimination of prostate cancer on one hand, or ovarian, endometrial, and (generally) breast cancer on the other. The genes identified in the top-20 feature group might indicate different genetic patterns with respect to primary sites. For example, *APC* and *KRAS*, the genes with the greatest importance scores, have already been widely researched where their role in carcinogenesis is concerned. *APC* loss-of-function mutations are common occurrences in colorectal cancer [17]. For this reason, this is likely a key feature for segregating metastatic colorectal cancer from the other classes. On the other hand, *KRAS* gain-of-function mutations are associated with not only colorectal cancer but also pancreatic adenocarcinoma and non-small cell lung cancer [18]. This likely gives the feature discriminatory power where lung and hepatobiliary cancers are concerned. Moreover, it is known that these two genes can have a synergistic effect through activating the Wnt and PI3K-AKT signaling pathways, which are both implicated in enhancing HIF-1α expression, chemotherapy resistance, and increases in metastatic potential [19,20,21]. By observing relationships between other genes listed as significantly important, perhaps in terms of their downstream signaling pathways, new mutational combinations with oncological relevance might be discovered.

### 3.4. Practical Applications of OncoOrigin in Precision Oncology

The possibility of tumor sequencing and primary cancer site prediction in CUP cases holds the potential for significant treatment benefits in the context of targeted oncological therapy. Precision medicine has revolutionized oncological therapy by enabling the implementation of targeted treatments based on specific molecular biomarkers of tumors [22]. Modern molecular biology techniques, such as whole genome sequencing (WGS) and whole exome sequencing (WES) of tumor tissue, facilitate the determination of the molecular profile of tumors and the application of therapies that act on specific molecular targets within the tumor tissue [7]. A key tool in this approach is tumor molecular profiling, which involves the genetic analysis of tumor tissue embedded in paraffin and/or circulating tumor DNA (ctDNA) obtained from liquid biopsy samples. The analysis of ctDNA from a patient’s blood represents a significant advancement in oncology, especially in cases where traditional tissue biopsies are not feasible [23]. These advancements in oncological diagnostics form the basis for the introduction of molecular classification of neoplasms aimed at targeted treatment of secondary tumors of unknown primary origin. The further development of the OncoOrigin platform, in addition to precise detection of the primary tumor site, opens up extensive possibilities for the molecular classification of secondary neoplasms based on results obtained through NGS methods. This advancement will pave the way for the diagnosis and precise treatment of CUP. Our planned further research will validate *in vivo* data obtained from the genetic analysis of tumor tissue embedded in paraffin and ctDNA derived from liquid biopsy samples. Should the quality of the OncoOrigin model hold its standard when tested on data obtained from liquid biopsies, we would open the possibility for early detection and treatment of metastatic cancers without relying on standard invasive methods such as surgery and PET-CT scans, which expose patients to additional ionizing radiation that can damage healthy cells [3]. Furthermore, in the case that more than one primary cancer site is likely, our software offers this information to the clinician. As multiple primary cancers can be managed with a combined regimen, this can in certain cases be actionable information [24].

## 4. Materials and Methods

### 4.1. Study Design

**Figure 5:**
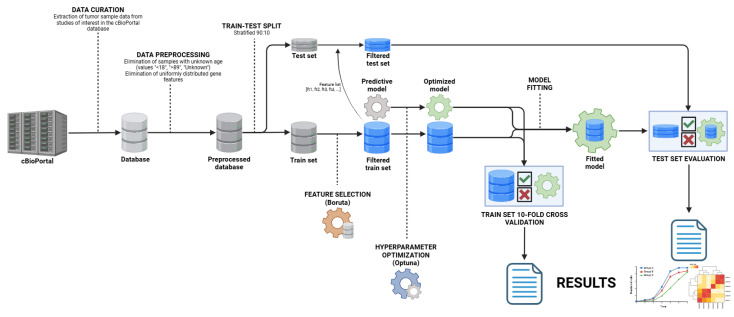
Data curation and model development flow chart (created with Biorender.com, accessed on 21 September 2024).

By design, this was an *in silico* study that involved the curation, processing, and utilization of publicly available data to develop a new machine learning model (Figure 5).

All of the data and code used in this study are publicly available (stated in the Data sharing statement) and the study design was approved by an institutional ethics committee (stated in the Ethical statement).

### 4.2. Data Curation

**Figure 6:**
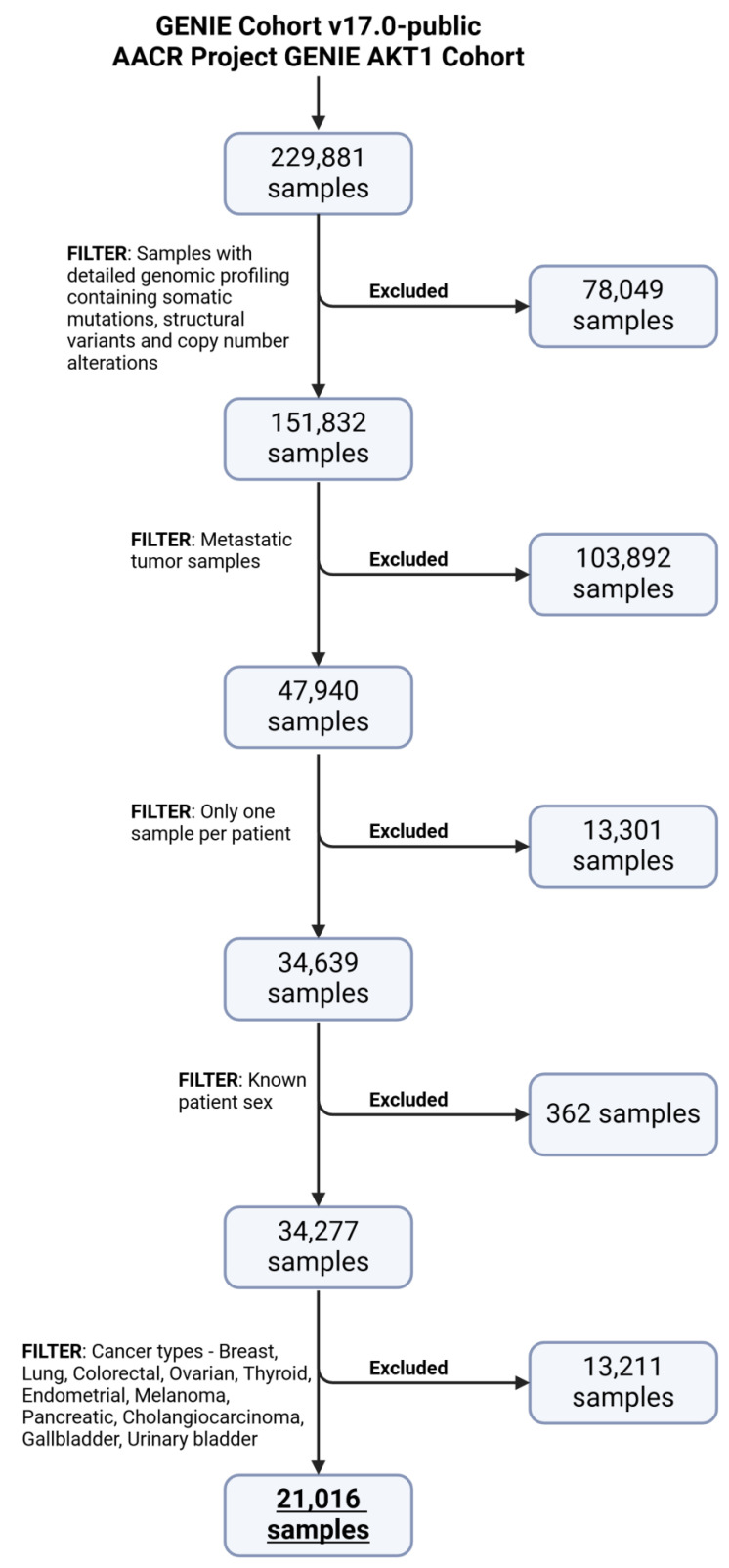
Data filtering process in the cBioPortal system (created with Biorender.com).

The data were extracted from cBioPortal (https://www.cbioportal.org/, accessed on 21 September 2024), a comprehensive database for cancer genomics [25,26,27]. cBioPortal contains clinical and genomic data from the AACR Project GENIE^®^ (Genomic Evidence Neoplasia Information Exchange). AACR Project GENIE (https://genie.cbioportal.org/, accessed on 21 September 2024) is a publicly accessible registry of real-world genomic data created through collaboration between 19 leading international cancer centers [28]. With approximately 287,000 sequenced samples from over 191,000 patients, it is one of the largest publicly available cancer genomic datasets, offering a vast resource for understanding the molecular underpinnings of various cancers. From this extensive dataset, 21,016 samples were selected through a rigorous filtering process for use in machine learning model development. The filtering process involved key criteria to refine the dataset (Figure 6).

Only one sample per patient was included to avoid multiple sequencing events for the same metastatic tumor. The selection included 62.3% female (13,085 samples) and 37.7% male (7931 samples) patients. The patient’s age at the time of sequencing was also utilized from the dataset. The virtual study created included 19 different subtypes of metastatic tumors.

Data for 665 genes were extracted for each sample, selected according to previously published validated panels as part of the implementation of whole-exome sequencing for tumor tissue analysis [2]. For the processing and utilization of data on single nucleotide variants (SNVs), copy number variations (CNVs), and structural variants to develop machine learning models, a binary system was employed. The presence of an SNV, CNV, or structural gene alteration was coded as 1, while the absence of these changes was coded as 0.

### 4.3. Data Preprocessing

Initial preprocessing included elimination of samples with unknown patient age (classified as “Unknown”, “<18”, or “>89”) and samples with Not-a-Number (NaN) values. Furthermore, gene features with no detected variants across all samples were eliminated. Additionally, the “Hepatobiliary Cancer” and “Pancreatic Cancer” classes were merged into the “Hepatobiliary/Pancreatic Cancer” class and the “Small Cell Lung Cancer” and “Non-Small Cell Lung Cancer” classes were merged into the “Lung Cancer” class. The output vector consisted of ten classes, “Colorectal Cancer”, “Ovarian Cancer”, “Melanoma”, “Breast Cancer”, “Lung Cancer”, “Thyroid Cancer”, “Prostate Cancer”, “Hepatobiliary/Pancreatic Cancer”, “Bladder Cancer”, and “Endometrial Cancer”.

### 4.4. Model Development

Four robust ensemble/gradient boosting machine learning algorithms, based on decision trees, were used for the prediction of cancer site—RandomForestClassifier (RFC), XGBoostClassifier (XGBC), CatBoostClassifier (CBC), and ExtraTreesClassifier (ETC). The preprocessed database was initially split into training and test sets at a 90:10 size ratio, using stratification with respect to the predicted class (cancer site). Feature selection among the gene features was conducted on the training set, using the Boruta algorithm with an optimized XGBC estimator. Secondly, hyperparameter optimization based on the train set was performed for each of the four algorithms using the Optuna library. The criterion for optimal hyperparameter identification was a sum metric using accuracy, F1, and ROC-AUC, calculated through 10-fold cross-validation in each trial. Finally, the models were initialized with optimal hyperparameters and fitted with the filtered training set. After the evaluation stage, the best estimator was chosen and fitted with the entire preprocessed database. This model was used for the development of the OncoOrigin software and feature importance analysis.

### 4.5. Model Evaluation

After fitting, each of the estimators was evaluated based on several metrics. All metrics were determined using the one-versus-rest setting for multiclass classification. The first step of evaluation was stratified 10-fold cross-validation on the training set after feature selection and hyperparameter optimization. In this step, mean accuracy scores, mean weighted F1 scores, and mean weighted receiver operating characteristic area under curve (ROC-AUC) scores were determined from each of the ten repetitions. Additionally, 95% confidence intervals were determined for these metrics. The second step involved performance evaluation on the segregated test set. For this analysis, accuracy scores, top-2 accuracy scores, weighted F1 scores, weighted ROC-AUC scores, weighted precision–recall area under curve (PR-AUC) scores, and class-specific sensitivity and specificity values were determined. Top-2 accuracy represents accuracy but the prediction is considered correct if the true value is one of the two most probable predicted classes. Additionally, confusion matrices and class-specific PR and ROC curves were determined for each estimator based on test set evaluation.

### 4.6. User Interface Development

**Figure 7:**
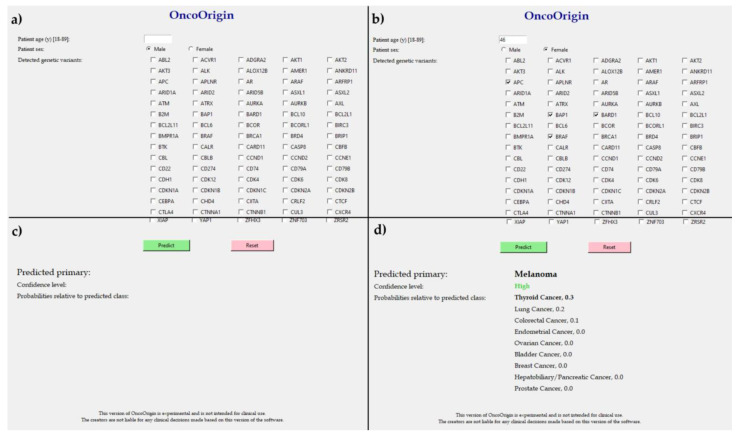
OncoOrigin graphical user interface with initial window (**a**), inputted patient age, sex, and genes with detected variants (**b**), bottom of the window with predict function (**c**), and prediction report with predicted site, confidence, and relative probabilities for other sites (**d**).

The final fitted XGBC model was exported and packaged into software with a graphical user interface (GUI)—OncoOrigin (Figure 7).

In the software, the user input consists of the patient’s age at time of sequencing, the patient’s sex, and genes in which genetic variants were discovered after tumor sequencing. The interface outputs the predicted class, as well as prediction confidence and probabilities for all other classes relative to the predicted class. The prediction confidence is determined by the relative probability *R_i_*. For a given class, *R_i_* is calculated as *p_i_*/*p*_0_, where *p_i_* is the absolute probability of a given class and *p*_0_ is the absolute probability of the predicted class. The level of confidence was determined by the relative probability *R*_2_ of the second most probable class (*R*_2_ ≥ 0.8 for very low, 0.8 > *R*_2_ ≥ 0.6 for low, 0.6 > *R*_2_ ≥ 0.4 for moderate, 0.4 > *R*_2_ ≥ 0.2 for high, and *R*_2_ < 0.2 for very high).

### 4.7. Programming Implementation

All steps involving machine learning model training and evaluation, as well as GUI development, were carried out in the Python/Jupyter Notebook programming language (v3.12.3) [29]. Several Python libraries were used, including Pandas (v2.2.2), NumPy (v1.26.0), MatPlotLib (v3.8.0), Seaborn (v0.13.2), SciPy (v1.14.0), Scikit-Learn (v1.5.1), XGBoost (v2.1.0), CatBoost (v1.2.5), Boruta (v0.3), and Optuna (v3.6.1) [30,31,32,33,34,35,36,37,38,39]. Development of the OncoOrigin GUI was done using the Tkinter (v8.6.13) library [40]. Random state variables throughout the code were defined by a chosen value. Still, due to inherent non-determinism in some of the stated libraries, different runs of the code did not produce the exact same results. However, noticed deviations in evaluation metric values due to non-determinism were negligible.

## 5. Conclusions

Precision medicine has revolutionized oncology by enabling targeted treatments based on molecular tumor profiling. Whole genome sequencing (WGS) and whole exome sequencing (WES) have significantly advanced tumor classification, allowing for personalized therapy approaches, particularly in diagnostically challenging cases such as cancer of unknown primary (CUP). Given the poor prognosis of CUP patients and the limitations of traditional diagnostic methods, innovative approaches that integrate molecular profiling with artificial intelligence are crucial for improving diagnostic accuracy and therapeutic outcomes.

The results of this study demonstrate that OncoOrigin is a robust tool for primary cancer site identification with strong potential for clinical application, particularly in CUP cases. By combining genetic data from over 20,000 tumor samples with machine learning algorithms, OncoOrigin offers a novel approach to molecular tumor classification. Unlike conventional models, OncoOrigin not only predicts the primary tumor site but also provides probability scores for multiple potential sites, offering clinicians more comprehensive and actionable information.

### Future Directions

Although OncoOrigin demonstrated excellent performance *in silico*, further validation is necessary before its implementation in clinical practice. The next step involves a multi-centric evaluation on patients with metastatic cancer of known primary origin, enabling real-world assessment of its predictive accuracy. If successful, the subsequent phase will focus on CUP cases, evaluating the clinical impact of OncoOrigin in guiding personalized therapy strategies.

Looking ahead, the integration of OncoOrigin with liquid biopsy technologies could further transform oncology diagnostics. By analyzing circulating tumor DNA (ctDNA), the software could potentially identify the primary tumor site without the need for invasive procedures. This could significantly enhance early detection, reduce diagnostic delays, and improve treatment precision. Ultimately, our goal is to position OncoOrigin as a key tool in precision oncology, offering a new standard for CUP diagnosis and contributing to a paradigm shift in clinical oncology.

## Figures and Tables

**Table 1:** Evaluation metrics for each estimator.

Metric		XGBC	CBC	ETC	RFC
**TRAIN SET 10-FOLD CROSS-VALIDATION**					
Mean accuracy		0.79	0.78	0.55	0.37
Accuracy 95-CI		0.782–0.793	0.771–0.784	0.543–0.561	0.365–0.375
Mean weighted F1		0.79	0.78	0.54	0.23
Weighted F1 95-CI		0.781–0.791	0.769–0.782	0.533–0.552	0.225–0.232
Mean weighted ROC-AUC		0.97	0.96	0.90	0.87
Weighted ROC-AUC 95-CI		0.965–0.968	0.962–0.966	0.895–0.902	0.868–0.877
**TEST SET EVALUATION**					
Accuracy		0.81	0.80	0.57	0.37
Top-2 accuracy		**0.91**	0.90	0.74	0.47
Weighted F1		0.81	0.80	0.56	0.23
Weighted ROC-AUC		**0.97**	0.97	0.91	0.88
Weighted PR-AUC		0.88	0.87	0.71	0.63
Sensitivity	Colorectal Cancer	0.86	0.86	0.79	0.00
	Ovarian Cancer	0.64	0.64	0.68	0.00
	Melanoma	0.86	0.85	0.66	0.00
	Breast Cancer	0.88	0.88	0.53	0.94
	Lung Cancer	0.79	0.77	0.15	0.74
	Thyroid Cancer	0.70	0.67	0.77	0.00
	Prostate Cancer	0.92	0.93	0.90	0.00
	Hepatobiliary/Pancreatic Cancer	0.81	0.79	0.82	0.00
	Bladder Cancer	0.38	0.33	0.50	0.00
	Endometrial Cancer	0.61	0.54	0.76	0.00
Specificity	Colorectal Cancer	0.98	0.98	0.97	1.00
	Ovarian Cancer	0.97	0.97	0.93	1.00
	Melanoma	0.99	0.98	0.97	1.00
	Breast Cancer	0.95	0.94	0.98	0.66
	Lung Cancer	0.95	0.94	1.00	0.53
	Thyroid Cancer	0.99	0.99	0.94	1.00
	Prostate Cancer	0.99	0.99	0.95	1.00
	Hepatobiliary/Pancreatic Cancer	0.97	0.97	0.91	1.00
	Bladder Cancer	1.00	1.00	0.96	1.00
	Endometrial Cancer	1.00	1.00	0.94	1.00

RFC—RandomForestClassifier, XGBC—XGBoostClassifier, CBC—CatBoostClassifier, ETC—ExtraTreesClassifier, ROC-AUC—receiver operating characteristic area under curve, PR-AUC—precision–recall area under curve, 95-CI—95% confidence interval, **0.xx**—highlighted metric, the optimal model (XGBC) metrics have been highlighted with a green background.

## Data Availability

The data used in this study are publicly available as part of a virtual study in the cBioPortal system, available at the following link: https://genie.cbioportal.org/study?id=670d2529854f636a3863065d (accessed on 21 September 2024). The source code files used for data management, model training and evaluation, and user interface development are publicly available as part of an active GitHub repository, available at the following link: https://github.com/lbulic1003/OncoOrigin (Source code/v1.0.0) (made available on 17 October 2024).

## Supplementary Materials

The following supporting information can be downloaded at: https://www.mdpi.com/article/10.3390/ijms26062568/s1.
